# Restricting O-Linked Glycosylation of the Mucin-like Domains Enhances Immunogenicity and Protective Efficacy of a Respiratory Syncytial Virus G Glycoprotein Vaccine Antigen

**DOI:** 10.3390/vaccines13101004

**Published:** 2025-09-25

**Authors:** Sara M. O’Rourke, Jackelyn Murray, Maria G. Juarez, Ralph A. Tripp, Rebecca M. DuBois

**Affiliations:** 1 Department of Biomolecular Engineering, University of California Santa Cruz, Santa Cruz, CA 95064, USA; sorourke@ucsc.edu; 2 Department of Infectious Diseases, University of Georgia, Athens, GA 30605, USA; 3 Department of Molecular, Cell, and Developmental Biology, University of California Santa Cruz, Santa Cruz, CA 95064, USA

**Keywords:** RSV, vaccine, recombinant RSV G^ecto^, glycoprotein, O-linked glycosylation, immunogen

## Abstract

**Background:** As of 2024, three approved respiratory syncytial virus (RSV) vaccines are licensed for use in adults in the United States: Arexvy™, Abrysvo™, and mRESVIA™. These vaccines are specifically designed to prevent lower respiratory tract disease caused by RSV in adults aged 60 and older. All licensed vaccines rely on stabilized RSV pre-fusion F (pre-F) as the sole antigen. RSV vaccines targeted to the other key RSV surface protein, the G glycoprotein, have been slow to advance because of sequence diversity and a historical association with vaccine-enhanced disease in animal models of infection. The recent development of structure-guided subunit immunogens and immune-modulating adjuvants has renewed interest in RSV G, as the combination of both F and G glycoproteins appears to improve vaccine efficacy over either one individually. RSV G is extensively O-glycosylated, with two mucin-like regions. **Methods:** This study investigated the effects of manipulation of O-linked glycosylation on a recombinant RSV G vaccine antigen in an RSV/A2 challenge study in BALB/c mice. **Conclusions:** We found that restricting the O-linked glycosylation on a recombinant RSV G vaccine antigen enhances its immunogenicity and protective efficacy in BALB/c mice.

## 1. Introduction

Respiratory syncytial virus (RSV) infection is the major cause of lower respiratory tract disease in infants and young children and the elderly. In 2019, 33 million RSV-associated acute lower respiratory infections and 3 to 6 million RSV-associated hospital admissions were recorded worldwide [1]. Recently, three adult-use vaccines have been approved in the United States: Abrysvo (Pfizer), Arexvy (GSK), and mRESVIA (Moderna). All licensed vaccines are focused on the F glycoprotein primarily because it is more conserved than the G glycoprotein [2]. The Abrysvo vaccine offers up to 70% protection from severe RSV disease in infants of immunized mothers up to 180 days after birth, but this response falls to ~40% efficacy after the infant reaches six months of age [3]. Additionally, all three vaccines offer protection against severe RSV disease in the elderly population, with efficacies ranging from approximately 62% to 84% [4,5,6].

The RSV G attachment glycoprotein mediates viral attachment to cells and modulates host immunity [7,8,9]. The G glycoprotein consists of an extracellular region that consists of two highly variable mucin-like domains, and a central conserved domain (CCD) of approximately 40 amino acids [2]. RSV has two major subgroups, A and B, defined by the G glycoprotein sequence. RSV/A tends to be more prevalent globally and may be linked to more severe disease. RSV/B can co-circulate simultaneously and may dominate in different regions or seasons. Genetic differences between subgroups are significant, with numerous distinct genotypes within each group, which influence their prevalence and spread [10]. The RSV G mucin-like domains 1 and 2 display strain-specific epitopes [11,12], and their mucin type O-glycosylation, which is highly diverse in nature and function, may impact both protein folding and immunogenicity [13,14]. Within the central conserved domain is a CX3C motif (aa182–186) that mimics the cysteine signature found in the chemokine CX3CL1 [15]. Soluble CX3CL1 is an immune-cell-recruiting chemokine that binds to the CX3CR1 receptor, while a membrane-bound version of CX3CL1 promotes cell adhesion [16]. Engagement of CX3CR1 on ciliated airway epithelia by the RSV G glycoprotein facilitates viral attachment and subsequent infection [17,18,19,20].

Human infant neonatal B regulatory cells are also infected via the CX3CR1 receptor, and upregulated expression of CX3CR1 correlates with disease severity [21]. Although the biological function of the RSV G protein mucin-like domains and the benefit of antibodies targeting these regions is still unclear, over the last 10 years, sequence duplications have been observed in the second mucin domain of circulating RSV A and B strains [22,23].

There has been a historical hesitance to explore the use of the RSV G glycoprotein as a vaccine antigen, primarily due to its extensive mucin-like domain sequence variability, low immunogenicity, and the potential, for dysregulation of CX3CL1-mediated CX3CR1 host immune interactions [15,24,25,26,27,28,29,30,31]. Nevertheless, structure-guided immunogen design and novel adjuvants have revived interest in G as a vaccine antigen. Monoclonal antibodies targeting the CCD block the interaction of RSV G glycoprotein with CX3CR1 [32,33,34,35,36,37] and are protective against inflammatory disease in mice [38,39,40,41,42,43,44,45] and cotton rat models of RSV infection [46]. Several studies have shown that antibodies to both F and G surface glycoproteins are associated with protection against RSV disease and may act synergistically [35,47,48,49,50,51,52]. A combination of both F and G glycoproteins appears to improve vaccine efficacy over either one individually [38,39,41,46,48,51,52,53], and the complementary use of Toll-like receptor TLR-4 and TLR9 agonist adjuvants, or T-cell-blocking molecules such as Cyclosporine A, has improved vaccine safety [54,55,56,57,58].

RSV infection of different mammalian epithelial-like cell lines produces RSV G glycoprotein with differing glycosylation and antigenic properties [59]. Recombinant mammalian-cell-produced RSV G ectodomain (G^ecto^) comprises greater than 60% by mass of oligosaccharide, the majority in the form of O-linked glycans that densely mask the protein backbone of the mucin-like domains. In contrast, the immunodominant conserved central domain is non-glycosylated. RSV G glycoprotein derived from virus-infected human airway epithelial cells likely comprises substantially more oligosaccharide [60] than that produced in commonly used mammalian cell lines. Glycosylation can facilitate protein folding, protein solubility, protease resistance, pattern recognition, epitope masking, immune evasion, and, in some cases, viral entry and egress, but the biological function of the mucin-like domains on the RSV G glycoprotein is yet to be fully elucidated [61,62]. Non-glycosylated *E. coli*-expressed RSV G^ecto^ was shown to be more immunogenic in mice than mammalian cell-produced RSV G^ecto^ containing glycosylation. While the latter induced significant lung pathology, non-glycosylated RSV G^ecto^ did not. Non-glycosylated *E. coli*-expressed RSV G^ecto^ also demonstrated a robust protective response in mice vaccinated with recombinant RSV G^ecto^ in combination with a CpG TLR9 agonist [57]. In these studies, no vaccine-induced lung pathology was observed. Furthermore, a recombinant non-glycosylated RSV G protein-based vaccine (BARS13) has just completed first-in-human randomized, double-blind, placebo-controlled dose-escalation phase II clinical trials, evaluating safety and immunogenicity in healthy adults aged 18–45 [55,63]. This protein-based vaccine is an *E. coli*-expressed recombinant RSV G^ecto^ administered in combination with Cyclosporine A. While the *E. coli* expression strategy was to eliminate glycosylation modifications that may function as a “glycan shield” for immune evasion, there are examples where glycosylation modifications play a nuanced but beneficial role in immunogenicity and vaccine design. For example, antibody recognition of specific high-mannose moieties provided an unexpected site of vulnerability in the HIV envelope glycoprotein [64]. This allowed the development of a new generation of HIV vaccine antigens [65] and potent neutralizing antibodies [66]. O-linked glycosylation is less well studied, but the recent characterization of the O-glycopeptidome [67], and identification of viral O-GalNAc peptide epitopes [13,61], is enlightening the field. For example, O-linked N-Acetyl galactosamine (GalNAc) is immunogenic and, due to its small size, may constitute a proteoglycan B-cell epitope absent on a non-glycosylated protein [68].

To explore the potential to enhance immunogenicity by restricting, but not completely excluding, O-linked glycosylation on an RSV G^ecto^ vaccine antigen, we performed a preclinical study using three Chinese Hamster Ovary (CHO) cell lines to express and purify RSV G^ecto^ with an identical amino acid sequence, but differing O-linked glycosylation. We selected the wild-type cell line, CHO-S, which generates simple mono- or di-sialylated O-glycosylation modifications, and two engineered CHO cell lines: (1) SimpleCell [69,70,71], which restricts O-linked glycosylation to the addition of the innermost N-acetylgalactosamine (GalNAc) on permissive serine and threonine amino acids, and (2) SimpleCell GALNT3, a SimpleCell line that expresses polypeptide N-acetylgalactosaminyltransferase 3 (GALNT3), which is otherwise absent in CHO cells in [72,73]. We chose to use the GALNT3 knock-in CHO line as polypeptide N-acetylgalactosaminyltransferase isoenzymes have partially overlapping but different peptide specificities [74], and down-regulation of GALNT3 is known to severely inhibit O-glycosylation of some mucins, such as the Muc10 protein [75]. Proteins were analyzed for appropriate folding by size exclusion chromatography and reactivity to conformation-dependent antibodies.

BALB/c mice were vaccinated with the three RSV G^ecto^ protein antigens, along with monophosphoryl-lipid A (MPLA) adjuvant, and then challenged with autologous RSV. We selected the MPLA as an adjuvant as it has been reported to engage the TLR4 receptor and promote a Th1-type response [76]. Vaccinated groups were tested for markers indicative of a protective response, such as specific antibody titer, neutralizing antibody induction, and lung viral load at peak infection. Additionally, we examined the IgG1 and IgG2a levels in serum and the cellular composition of bronchoalveolar lavage fluid [77].

## 2. Materials and Methods

### 2.1. Cell Lines

FreeStyle CHO-S cells were purchased (R80007, Thermo Fisher Scientific, Waltham, MA, USA). SimpleCell CHO SimpleCell GALNT3 CHO cell lines kindly provided by Dr. H. Claussen and Dr. Y. Narimatsu from the University of Copenhagen, Denmark. SimpleCells were generated by Zinc Finger Nuclease modification of an original CHO line deposited at the European Collection of Authenticated Cell Cultures as product number 85,051,005 via Sigma Aldrich (Burlington, MA, USA), as described in detail in Yang et al. 2014 [71]. The resulting CHO glycoproteome derived from analysis of these cells is described in the O-Glycoproteome DB: http://glycoproteomics.somee.com. The GALNT3 “knock in” protocol is described in [73,78]. Freestyle HEK293F cells were purchased (R79007, Thermo Fisher Scientific, Waltham, MA, USA). Hep2 cells (CCL-023), A549 cells (CCL-185) and Vero cells (CCL-81) were purchased from the American Type Culture Collection (ATCC, Manassas, Virginia, USA). Hep2 cells were used to propagate viruses, and Vero cells for plaque assay. Neither line is susceptible to anti-RSV G-mediated neutralization [79]; therefore, the human lung adenocarcinoma epithelial cell line (A549) was used for microneutralization assay as described below. All cell lines were immediately frozen on receipt, used at low passage number and tested negative for mycoplasma contamination. Murine mAbs 143-6C and clone 131-2A were isolated from hybridomas described in [80], kindly provided to the Tripp Lab by Dr. L. Anderson of Emory University (Atlanta, GA, USA).

### 2.2. Immunogens

Recombinant RSV G^ecto^ proteins were produced in serum-free suspension cultures of CHO-S, SimpleCell and SimpleCell GALNT3 CHO cells. A codon-optimized synthetic gene encoding RSV strain A2 (RSV/A2) G glycoprotein amino acids 64–298 (UnitProtKB entry P03423) from Genscript (Piscataway, NJ, USA) was cloned into a pcDNA3.1 vector optimized for expression, with an N-terminal CD5 signal peptide, and tandem C-terminal 6× His-tag and Twin-StrepTactin purification tags. For expression, endotoxin-free plasmid was introduced into each cell line via electroporation, and stable pools were selected based on G418 resistance conferred by the Neomycin resistance gene from Tn5 encoding an aminoglycoside 3′-phosphotransferase, APH 3′ II. Media were harvested and processed for protein purification as previously described [65,80,81,82]. Secreted RSV G^ecto^ protein was affinity purified on a StrepTactin XT column (Cytiva, Marlborough, MA, USA), followed by size-exclusion chromatography on a Superose-6 increase 10-300 column (Cytiva). All proteins were concentrated to 1 mg/mL, dialyzed in PBS, flash-frozen in liquid nitrogen, and stored at −80 °C until use. The protein purity was evaluated via sodium dodecyl sulfate-polyacrylamide gel electrophoresis (SDS-PAGE), size-exclusion chromatography, and anti-His-tag Western blot analysis. Freestyle HEK293F were cultured according to the manufacturer’s protocol, in Freestyle media (Thermo Fisher Scientific, Waltham, MA, USA).

### 2.3. Monoclonal Antibodies (mAbs) and Recombinant CCD-Fc Protein

The anti-RSV G glycoprotein monoclonal antibody (mAb) 2D10, was expressed by transient transfection of CHO-S cells and purified from CHO-S conditioned media as previously described [82]. MAb 3D3 was from Trellis Bioscience (Redwood City, CA, USA). Murine mAbs 143-6C and clone 131-2A [80] were isolated from cultured hybridomas. To measure serum antibodies against the central conserved domain (CCD) alone, amino acids a synthetic gene 157–198 (Genscript) was cloned in-frame with a human Fc domain in a plasmid previously used for expression of CX3CL1 chemokine domain fused to Fc [83] using Gibson Assembly. The CCD-Fc fusion has an N-terminal murine kappa-light chain secretory signal and a C-terminal 6× His-Tag. Recombinant CCD-Fc was produced from G418-resistant HEK293F transfected cells, purified using His-Trap Excel column chromatography (Cytiva), and dialyzed in PBS before flash freezing for storage at 1 mg/mL at −80 °C. Accessibility of the CCD was assessed by measuring the affinity of the CCD-Fc protein to conformation-dependent Fab fragments of two anti-CCD antibodies, mAbs 2D10 and 3D3 [36].

### 2.4. Binding-Affinity Analysis

The binding affinities of O-glycan-restricted RSV G^ecto^ to mAbs 2D10 and 3D3 were determined using biolayer interferometry (Octet RED384, Sartorius, Freemont, CA, USA). Anti-human-Fc Capture (AHC) biosensors (Sartorius, 18-5060) were immersed in binding buffer (PBS pH 7.4, 1% bovine serum albumin, 0.05% Tween-20) for 60 s and then loaded sequentially with 1 µg/mL of antibody for 120 s, followed by 120 s in binding buffer. Association was measured with a 2-fold serial dilution series (20–5 nM) of G^ecto^ over 300 s and dissociation was measured over 600 s. A global 1:1 fit model was used to fit at least three concentrations of RSV G^ecto^ to calculate the equilibrium dissociation constant (K_D_). Average K_D_ values are reported as the average of the three replicates calculated from the dilution series.

### 2.5. Mice

Specific-pathogen-free, 6–8-week-old female BALB/c mice (Jackson Labs, Bar Harbor, ME, USA) were used in all studies. The mice were housed in microisolator cages (5 animals per cage), 5 animals per experimental group, 30 animals in total, and given ad libitum access to food and water. All experiments were performed according to the guidelines of the University of Georgia’s Institutional Animal Care and Use Committee (IACUC), following protocols approved by the University of Georgia IACUC (approval date: 5 May 2018; approval: A2018 04-018-A2). Mice were randomly assigned to treatment groups.

### 2.6. Virus

RSV/A2 was propagated in HEp-2 cells (American Tissue Culture Collection ATCC, CCL-023), as previously described [84]. The HEp-2 cell line contains HeLa marker chromosomes and were derived via HeLa contamination. Mice were anesthetized via intraperitoneal administration of 2,2,2-tribromoethanol (Avertin; 200 mg/kg) and intranasally challenged with 10^6^ plaque-forming units (PFUs) of RSV/A2 in 30 μL PBS (Hyclone, South Logan, UT, USA).

### 2.7. Vaccination

Mice were vaccinated intramuscularly with 50 μg of immunogen or PBS + 50 μg synthetic monophosphoryl lipid A (MPLA, InVivogen, San Diego, CA, USA), diluted in PBS. Mice (*n* = 5 per group) were primed on day 0 and boosted on day 21 via homogenous vaccination. On day 40 (19 days post-boost), the mice were intranasally challenged with 10^6^ PFUs RSV/A2 and subsequently euthanized on day 5 post-challenge.

### 2.8. Detection of Anti-RSV G^ecto^ and Anti-CCD Specific IgG by ELISA

Flat-bottom, high-binding 96-well ELISA plates (Corning, 3590) were coated overnight at 4 °C with 5 µg/mL of recombinant RSV G^ecto^ or CCD-Fc protein, diluted in PBS. The plates were washed three times with wash-buffer, PBS, pH7.4, 0.1% Tween (Accuwash, Thermo Fisher Scientific, Waltham, MA, USA), and blocked for two hours at 25 °C with Blotto (PBS pH7.4, 5% non-fat dry milk). Pre-challenge sera samples collected at Day 19 and Day 35 of the experiment were serially diluted 1:3 in Blotto (starting at a 1/40 dilution) and then incubated for two hours at 25 °C. After washing three times with wash buffer, the plates were incubated for one hour at 25 °C with a goat anti-mouse Fc-specific horseradish peroxidase (HRP)-conjugated secondary antibody (115-035-071, Jackson-ImmunoResearch, West Grove, PA, USA), diluted 1/5000 in Blotto. The plates were washed 3 times and developed for 10 min in the dark with TMB Substrate (Thermo Fisher Scientific, Waltham MA, USA), before stopping the reaction with 1 N H_2_SO_4_. The optical density at 450 nm, OD450, was read on a plate reader (Molecular Devices, San Jose, CA, USA). The resulting graphs are representative of two independent experiments. The background cut-off was calculated as the mean OD450 signal in the absence of primary antibody, but the presence of secondary antibody, plus three times the standard deviation, plus 10% of the calculated number.

### 2.9. Determining RSV Lung Titers by Plaque Assays

RSV lung titers in vaccinated and control mice were determined as previously described [76]. Briefly, lungs were aseptically removed from exsanguinated mice on day 5 after challenge, and individual lung specimens were placed in 1 mL Dulbecco’s modified Eagle’s medium and homogenized using a gentleMACS dissociator (Miltenyi Biotec, San Jose, CA, USA). After centrifugation of the extract at 400× *g* for 10 min at 4 °C, 200 µL/well, serial 10-fold dilutions of the soluble homogenate fraction were added to 90% confluent Vero cell (ATCC CCL-81) monolayers in 24-well tissue culture plates, allowing 2 h at 37 °C for viral absorption. The cell monolayers were overlaid with medium containing 2% methylcellulose and further incubated at 37 °C for 6 days. To enumerate plaques, the monolayers were fixed with 60% acetone/40% methanol, then incubated overnight at 4 °C with a 1 μg/mL of monoclonal antibody against the RSV F protein (clone 131-2A), followed by a further 1 h incubation at 25 °C with a 1/1,000 dilution of goat anti-mouse HRP-conjugated IgG antibody (Jackson ImmunoResearch West Grove, PA, USA) before visualizing with KPL TrueBlue peroxidase substrate (SeraCare, Milford, MA, USA).

### 2.10. Detection of Anti-RSV/A2 Antibodies by ELISA

An indirect ELISA specific to RSV/2 was examined using flat-bottom high-binding ELISA plates (Corning, 3590) that were coated with 10^6^ PFU/mL RSV/A2 was diluted in PBS (pH 7.4) and added to the wells for 1 h at 37 °C. The plates were washed three times with 1× KPL Wash Buffer (SeraCare Milford, MA, USA) and blocked for 2 h at 37 °C with 5% non-fat dry milk + 1% BSA (Sigma Aldrich, Burlington, MA, USA) diluted in 1× KPL wash buffer. The blocking solution was decanted, and the plates were incubated for 1h at 37 °C with sera diluted in 5% non-fat dry milk + 1% BSA. The solution was decanted, and the wells were washed 3x with KPL wash buffer. Subsequently, the wells were incubated for 1 h at 37 °C with a 1/5,000 dilution of an HRP-conjugated secondary goat anti-mouse antibody against murine Fc (Thermo Fisher Scientific, Waltham, MA, USA) or an HRP-conjugated secondary IgG1 or IgG2a goat anti-mouse antibody (Southern Biotech, Birmingham, AL, USA), diluted per the manufacturer’s instructions. The plates were washed in PBS (pH 7.4) and developed with 1-Step Ultra TMB Substrate (Thermo Fisher Scientific, Waltham, MA, USA). The reactions were stopped with Stop-Solution (Invitrogen). OD_450_ and values read using a plate reader (BioTek, Winooski, VT, USA). The data were plotted using GraphPad Prism software, version 10.4 (GraphPad, Boston, MA, USA), and error bars represent standard deviation.

### 2.11. Bronchoalveolar Lavage (BAL) and Flow Cytometry

To investigate the composition of BAL cells in vaccinated mice at day 5 post-challenge, lungs were washed 3x with 1 mL ice-cold PBS. BAL was centrifuged at 500× *g* for 5 min at 4 °C. BAL cells were washed and resuspended in FACS buffer (PBS with 1% BSA and 1 mM EDTA). Cell-surface marker expression patterns were used to identify CD3^+^ T cells, eosinophils (CD45^+^/SiglecF^+^/CD11c^low^), alveolar macrophages (CD45^+^/SiglecF^+^/CD11c^high^) and activated B cells (CD45^+^, CD220^+^). Cell suspensions were blocked with an anti-FcγII/III receptor antibody (BD Bioscience, Franklin Lakes, NJ, USA) and subsequently stained with fluorescent antibodies from eBiosciences (San Diego, CA, USA), BioLegend (San Diego, CA, USA), or BD Bioscience, i.e., PerCP-conjugated anti-CD45 (30-F11), APC-conjugated anti-CD11c (HL3 or N418), PE-conjugated anti-SiglecF (E50-2440), CD45^+^ CD220^+^ (clone RA3-6B2), FITC-conjugated anti-CD3e (145-2C11), per the suppliers’ instructions. The cells were analyzed on a Guava easyCyte 8 flow cytometers (Millipore, Burlington, MA, USA), and the data were analyzed using Guavasoft software (version 2.7 or 3.1.1). The cells were gated based on forward scatter and side scatter, and then by CD45^+^ staining. Eosinophils were identified as FSC^high^/SSC^high^/CD45^+^/SiglecF^+^/CD11c^low^ cells.

### 2.12. Microneutralization Assay

A microneutralization assay was used to assess anti-RSV-mediated neutralization in mouse serum samples [85]. Briefly, the sera were pooled and heat-inactivated at 55 °C for 30 min. The sera were diluted 1:40 in DMEM containing 2% fetal bovine serum and incubated for 1 h at 37 °C with 200 fluorescent focus units (FFUs) RSV/A2-GFP with or without 10% guinea pig complement (C’) (Sigma Aldrich, Burlington, MA, USA). Following pre-incubation, the virus/serum mixture was added to 95% confluent A549 (purchased from the ATCC, A549-CCL-185) and used at low passage number) and the cells were incubated for 48 h. Anti-RSV F protein neutralizing mAb 143.6C was included as a technical control. FFUs were visualized using a Cellomics ArrayScan (Thermo Fisher Scientific, Waltham, MA, USA), and the mean FFU value of replicate wells was determined using HCS Studio™ Cell Analysis Software (Thermo Fisher Scientific, Waltham, MA, USA, Scotts Valley, CA, USA). Neutralization was defined as the percent reduction in the mean FFU when compared with infection in the absence of serum.

### 2.13. Statistical Analysis

The challenge study was designed as a preliminary pilot experiment to investigate the experimental variation and attrition, as a guide to future larger experiments. No power calculation was performed. No animals were excluded from analysis. All statistical analyses were performed using GraphPad Prism software, version 10.4 (GraphPad Software). A single challenge study was performed. The data are presented as the mean ± standard deviation. After applying the Shapiro–Wilk normality test, differences between two groups were analyzed with one-way or two-way analysis of variance (ANOVA), where appropriate, followed by Dunnett’s multiple comparison test for group to control analysis, or Tukey HSD test for comparison among groups. *p* < 0.05 was considered statistically significant. Non-parametric tests were performed where indicated, using the Mann–Whitney U test (for two independent groups), the Kruskal–Wallis test (for more than two independent groups). Results of our statistical tests are indicated in the figure legends. For statistical analysis of flow cytometry, the data were analyzed by one-way ANOVA with Bonferroni’s correction or Student’s *t*-test for pairwise analysis.

## 3. Results

### 3.1. Expression and Purification of O-Glycan-Restricted RSV G^ecto^ Glycoproteins

**Figure 1:**
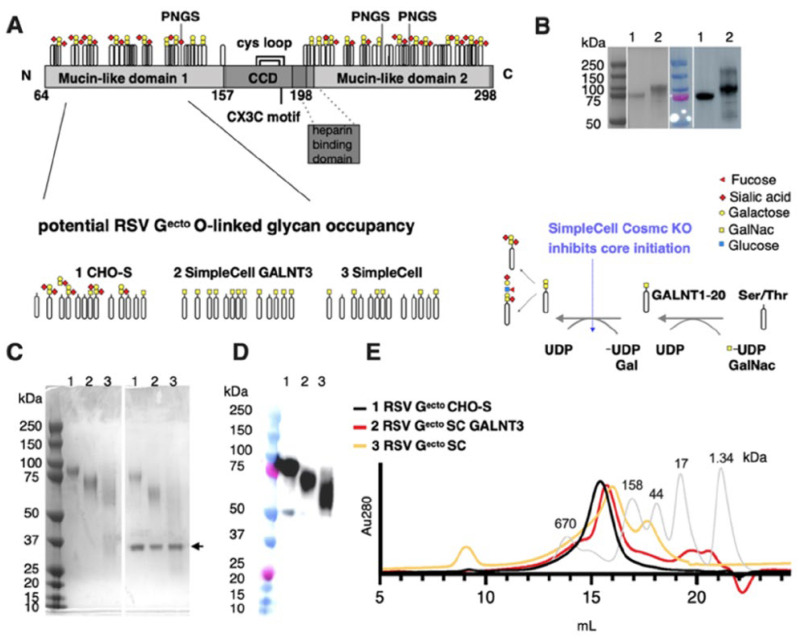
Evaluation of recombinant RSV G^ecto^ proteins produced in SC cell lines. (**A**) Schematic of recombinant RSV G^ecto^ comprising the conserved central domain (CCD), including its CX3C motif and cysteine loop, heparin-binding domain, and mucin-like domains 1 and 2, with a simplified depiction of potential O-linked glycosylation patterns and potential N-linked glycosylation sites (PNGS). O-glycosylation addition is initiated via polypeptide N-acetylgalactosaminyltransferases GALNT1-20. GALNT3 is not expressed in wild-type CHO cells. These transferases catalyze the addition of GalNac to Ser/Thr residues, permitting subsequent core synthesis, extension, and capping. In SimpleCell lines, core-1 synthesis is inhibited by the knockout of an essential chaperone, Cosmc, restricting O-glycosylation to the initial GalNAc on Ser/Thr residues. In SC GALNT3, GalNac is added to additional permissive Ser/Thr residues by GALNT3. (**B**) SDS PAGE followed by Coomassie stain and Western blot of RSV G^ecto^ purified from (1) CHO-S or (2) HEK 293 cells, with apparent molecular weights of ~90 and ~100 kDa, respectively. (**C**) SDS PAGE followed by Coomassie stain of RSV G^ecto^ purified from (1) CHO-S, (2) SC GALNT3, and (3) SC cells, with apparent molecular weights of ~90, 75, and 55 kDa, respectively. In the presence of PNGaseF (enzyme indicated by arrow), the bands downshift, indicative of the removal of N-linked glycans. (**D**) Western blot of recombinant RSV G^ecto^ with an anti-His-tag antibody. (**E**) Analytical size exclusion chromatography on a Superose 6 10-300 column of recombinant RSV G^ecto^ proteins (black, red, and yellow lines) compared to gel filtration standards (grey line).

RSV G glycoprotein from RSV/A2 is a 298 amino acid polypeptide, with three predicted N-linked glycosylation sites (PNGS) and between 30–40 predicted O-linked glycosylation sites, a transmembrane domain, and a short cytoplasmic tail. For the murine RSV challenge study, we produced protein immunogens corresponding to the mature ectodomain of RSV G (G^ecto^) that incorporates amino acids 64 to 298 (Figure 1A), and C-terminal tandem 6× histidine and Twin-StrepTactin purification tags, for detection and purification. RSV G^ecto^ protein produced in CHO cells has a slightly lower motility on an SDS-PAGE gel than RSV G^ecto^ protein produced in HEK 293 cells (Figure 1B). To generate O-glycan-restricted RSV G^ecto^ proteins, we used SimpleCell (SC) and SC GALNT3 CHO lines [69,70,71]. CHO-S, SC GALNT3, and SC lines produced RSV G^ecto^ proteins exhibiting apparent molecular masses of, 90 kDa, 75 kDa, and 55 kDa, respectively, by SDS-PAGE (Figure 1C), and PNGase F treatment resulted in a molecular mass reduction concomitant with stripping of the predicted 3 N-linked glycans (Figure 1C, panel 2) but having no effect on O-linked glycans. Interestingly, SC GALNT3 RSV G^ecto^ is ~20 kDa larger than SC RSV G^ecto^, which is due to increased occupancy by GalNAc, catalyzed by GALNT3 (Figure 1A). Western blot analysis with an anti-His-tag antibody confirmed that the peaks contained the recombinant RSV G^ecto^ proteins (Figure 1D).

Analytical-scale size exclusion chromatography of purified RSV G^ecto^ proteins resulted in a major peak eluting between the globular gel-filtration standards representing molecular weights of 670 and 158 kDa, with the CHO-S RSV G^ecto^ eluting at a single peak at around 15.5 mL. In contrast, SC GALNT3 and SC RSV G^ecto^ were retained longer, eluting at 16 and 16.5 mL, respectively (Figure 1E). Previous cryo-electron microscopy studies of the SC GALNT3 RSV G^ecto^ protein showed it to be monomeric, flexible, and having the capacity to simultaneously bind two conformation-dependent antibodies with high affinity [81].

### 3.2. Antigenicity of O-Glycan-Restricted RSV G^ecto^ Glycoproteins

To evaluate the effect of O-glycosylation restriction on RSV G^ecto^ protein folding, we determined the affinities of the RSV G^ecto^ proteins for two anti-RSV G CCD conformation-dependent monoclonal antibodies (mAbs) that bind to neighboring epitopes spanning the cysteine loop [36,81], by biolayer interferometry (BLI). In line with previous studies [36,81,82], mAbs 2D10 and 3D3 had low picomolar binding affinity for CHO-S RSV G^ecto^ (Table 1). Similar results were observed for SC-produced RSV G^ecto^. These studies reveal that the O-glycan-restricted recombinant RSV G^ecto^ retains conformational epitopes in the CCD.

### 3.3. Immunogenicity of O-Glycan-Restricted RSV G^ecto^ Protein Immunogens

**Figure 2:**
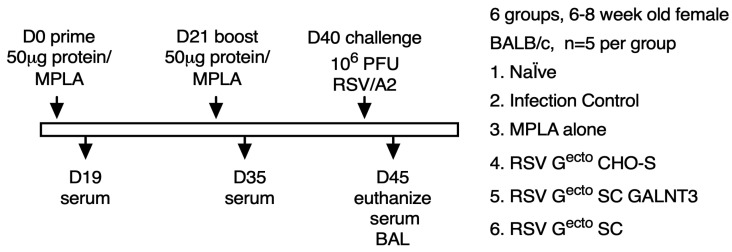
Outline of vaccination and challenge schedule. Six groups of BALB/c mice (*n* = 5) were primed with 50 µg of RSV G^ecto^ combined with 50 µg of MPLA, MPLA alone or PBS, and boosted at day 21 (D21) with the same vaccine antigen and adjuvant combination. Serum samples were assayed 19 days after priming at D19 and 14 days after homologous boosting at D35. Animals were subjected to intranasal challenge at D40, five days after boost, with 10^6^ PFU RSV/A2 virus, then euthanized five days later. Serum, BAL, and lung tissue were collected for analysis.

A 50 µg RSV G^ecto^ was combined with MPLA, an established TLR4 agonist. Six groups of 6–8-week-old female BALB/c mice were immunized intramuscularly in a prime-boost regimen and then challenged with RSV/A2, following the schedule shown (Figure 2).

**Figure 3:**
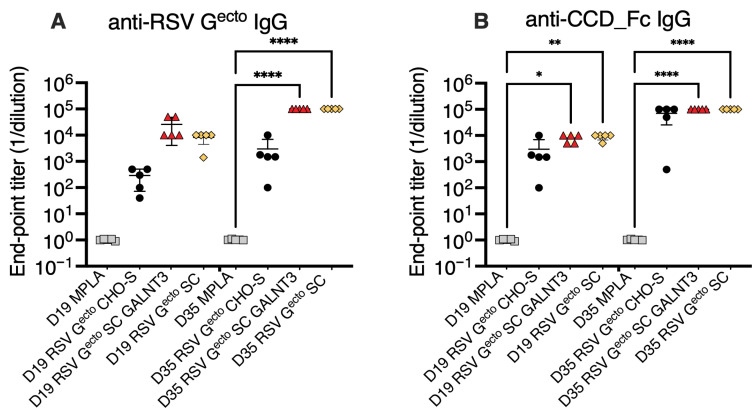
Comparison of non-restricted and O-glycan-restricted RSV G^ecto^ immunogenicity in mice. Circulating (**A**) Anti-RSV G^ecto^-specific IgG (**B**) and anti-CCD-Fc specific IgG were detected by an indirect-binding ELISA two weeks after prime (D19), and two weeks after boost (D35), with the three vaccine antigens (*n* = 5 mice per group) adjuvanted with MPLA or MPLA alone. Endpoint titer was calculated from a sigmoidal 5PL, where the *X*-axis is a log (serum dilution) plot with a cutoff value of baseline +3 times SD plus 10%. Groups were compared for statistical significance using a one-way ANOVA with Tukey’s post hoc correction, * *p* = 0.0226, ** *p* = 0.0069, **** *p* < 0.0001, 95% confidence.

To compare vaccine antigen immunogenicity, we calculated the endpoint titer of anti-RSV G^ecto^ and anti-CCD-specific IgG circulating after a single priming (D19), or the complete prime/boost regime (D35) by ELISA, as summarized in Figure 3. Neither anti-RSV G^ecto^ nor anti-CCD IgG was detected in the MPLA-alone group. Post-boost (D35), groups of mice vaccinated with recombinant O-glycan-restricted RSV G^ecto^ (SC GALNT3 and SC) antigens had significantly (*p* = 0.0001) higher anti-G^ecto^ endpoint titers (1/dilution) both at ~10^5^, compared to MPLA control group. Similarly, statistically significant anti-CCD-specific IgG endpoint titers of ~10^5^ (*p* > 0.0001), were only achieved post-boost in the O-glycosylation restricted RSV G^ecto^ vaccinated groups (Figure 3B).

Interestingly, although only approaching statistical significance for the RSV G^ecto^ SC GALNT3 vaccination (*p* = 0.0643), a single immunization (D19) elicited 10-fold more total anti-RSV-G^ecto^ IgG than the non-restricted O-glycosylated protein (Figure 3A). Measuring the anti-CCD specific IgG response after a single vaccination (D19), all mice vaccinated with O-glycosylation restricted RSV G^ecto^ adjuvanted with MPLA achieved a titer of 10^4^ (*p* = 0.0069 and *p* = 0.0226) compared to the baseline MPLA response (Figure 3B).

### 3.4. Evaluation of Post-RSV/A2 Challenge Responses

**Figure 4:**
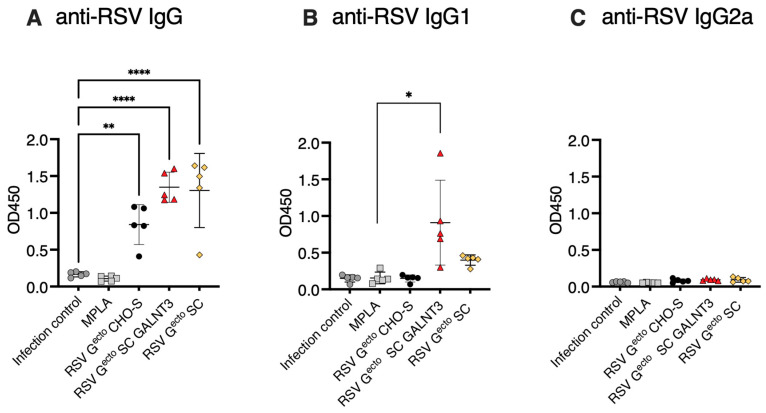
The IgG recall response of RSV/A2 challenged mice vaccinated with O-glycan restricted RSV G^ecto^ immunogens. Vaccinated mice (*n* = 5 per group) were intranasally challenged with 10^6^ PFU of RSV/A2. (**A**) Five days post-challenge, circulating anti-RSV-specific IgG was assayed by direct binding of antibodies in serially diluted serum samples to RSV/A2-coated ELISA plates. All antigens induced a significant anti-RSV/A2/specific IgG response when compared to the infection control (** *p* < 0.0031, and **** *p* < 0.001). OD_450_ (*Y*-axis) at single point dilution (1/1280) on the linear segment of the titration curve where OD values are proportional to amount of antibody, permitting comparison. Bars represent mean values and SD. (**B**) Relative amounts of circulating anti-RSV A2-specific IgG1, (**C**) IgG2a, were assessed at a single (1/40) serum dilution for mice in each challenge group. IgG1 was elevated in mice immunized with RSV G^ecto^ SC GALNT3 protein compared to the infection control (* *p* < 0.05). No group raised an IgG2a response above that observed in the infection control. Statistical testing was performed by a one-way ANOVA test using Tukey’s post hoc correction for (**A**) (as the dilution is on the linear part of the curve) and a Kruskal–Wallis test for (**B**,**C**).

RSV/A2-specific serum IgG antibody recall, serum neutralizing activity, and viral lung titer at the peak of infection were evaluated five days after intranasal challenge with 10^6^ PFU RSV/A2. All RSV G^ecto^-vaccinated groups had robust anti-RSV/A2 IgG recall responses, consistent with pre-challenge immunogen-specific titer (Figure 4A), further analyzed as IgG1 and IgG2a components in Figure 4B,C and discussed below. The group immunized with RSV G^ecto^ SC GALNT3 had a higher IgG1 response than the infection control (*p* < 0.05).

**Figure 5:**
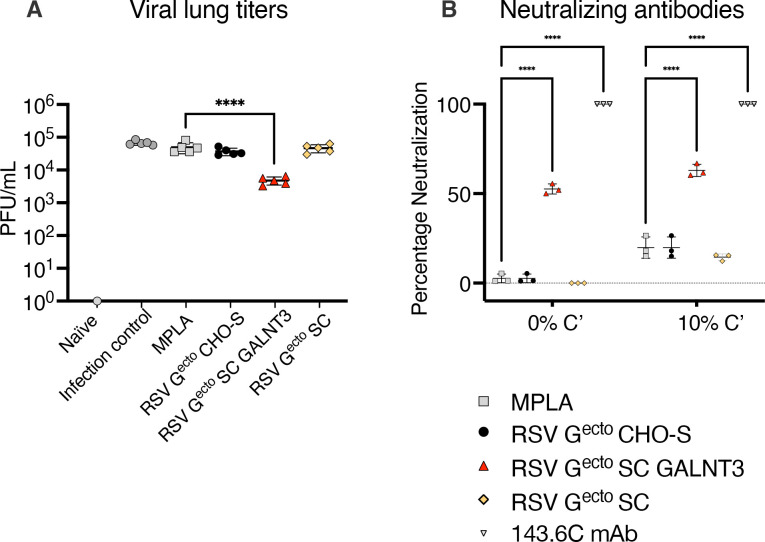
The lung viral load and neutralizing antibody response in BALB/c mice following vaccination with O-glycan restricted RSV G^ecto^ vaccines. (**A**) Viral lung titer/mL of lung homogenate as determined by plaque assay. Mice were immunized with O-glycan-restricted RSV G^ecto^ SC GALNT3 + MPLA. They had a significant (**** *p* = 0.0001) reduction in lung virus load (D45) at day 5 post-challenge compared to mice vaccinated with MPLA alone. (**B**) Pooled antisera and control anti-F glycoprotein neutralizing murine mAb 143.6C were heat-inactivated and diluted to 1/40 for microneutralization assay in A549 cells in the presence or absence of 10% guinea-pig complement. Fluorescent focus units (FFU) were enumerated on a Cellomics ArrayScan using HTS software. Bars represent mean ± SEM. Sera derived from RSV G^ecto^ SC GALNT3 protein vaccinated mice had a greater capacity to neutralize RSV A2 infection of A549 cells (**** *p* = 0.0001) than MPLA-treated controls. Bars represent mean ± SD. Group titers were compared by one-way ANOVA with Dunnett’s multiple comparison.

Lung viral load was quantified by plaque assay, and the data (Figure 5A) were summarized as PFU/mL of lung homogenate. A single vaccine immunogen, the O-glycan-restricted RSV G^ecto^ GALNT3 protein, conferred a significant reduction of mean viral load from 4.9 × 10^4^ PFU/mL in the mock vaccinated MPLA control group, to 4.8 × 10^3^ PFU/mL (*p* < 0.001). Finally, a significant (*p* = 0.0001) complement-mediated and classical (non-complement-dependent) neutralization of autologous RSV A2 virus was detected in the assay of sera from mice vaccinated with the O-glycan-restricted RSV G^ecto^ SC GALNT3 (Figure 5B) compared to MPLA controls.

### 3.5. Evaluation of Lung Biomarkers Associated with Vaccine-Enhanced Disease

The RSV G protein modulates host immunity by interfering with the CX3CL1/CX3CR1 interaction, raising questions about the safety of RSV G-based vaccines [7]. Host-mediated pathogenesis correlates with a Th2-type T-cell-mediated imbalance and cytokine cascade. Although the BALB/c murine infection model of infection is imperfect with respect to accurately reflecting human RSV infection [9,86,87,88,89], previous studies vaccinating BALB/c with fully glycosylated recombinant RSV G-derived proteins [28,57,90] have caused measurable increases in surrogate markers of vaccine enhanced disease such as inflammatory infiltrate, lung eosinophilia, cytokine profile and an imbalance in the ratio of RSV G protein specific IgG antibody subtypes.

**Figure 6:**
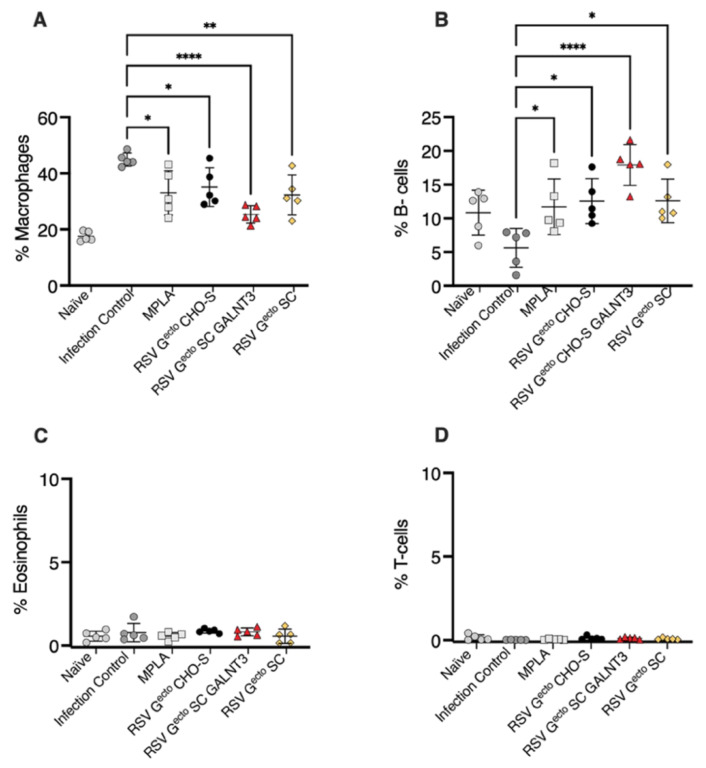
BAL leukocyte composition at day 5 in BALB/c mice with O-glycosylation restricted RSV G^ecto^ vaccines and challenged with 10^6^ PFU of RSV/A2. BAL composition by flow cytometry: charts represent the percentage of the total number of cells isolated with values for each vaccine control group compared to the infection control, applying a one-way ANOVA analysis with Tukey’s post hoc correction to compare groups. Bars represent mean ± SD. (**A**) Siglecf+ staining macrophages (CD45+/Siglecf+CD11c^low^) (* *p* = <0.05, ** *p* < 0.0056, **** *p* < 0.0001). (**B**) Percentage B220-positive B-cells (* *p* < 0.05, **** *p* < 0.0001). (**C**) Percentage Siglecf+ (CD45+/Siglecf+CD11c^low^) eosinophils. (**D**) Percentage positive CD3+ T-cells.

We evaluated the levels of serum anti-RSV/A2 IgG1 and IgG2a as a surrogate for a potential vaccine-induced Th2:Th1 imbalance. BALB/c mice vaccinated with O-glycan-restricted RSV G^ecto.^ SC GALNT3 had more RSV/A2-specific IgG1 than the adjuvant alone vaccinated mice (*p* = 0.05), and no vaccine antigen promoted the induction of an IgG2a response (Figure 4B,C). Examination of the BAL shown in Figure 6 did not indicate the presence of a pathological post-challenge vaccine-enhanced inflammatory infiltrate in the lungs of any of the groups of mice vaccinated with MPLA adjuvanted RSV G^ecto^. It is known that airway macrophages line the bronchiole membranes, promoting protection and homeostasis [91]. Still, the recruitment of activated macrophages following RSV challenge occurred in approximately 45% of the BAL leukocytes analyzed in the infection control. Typical BAL leukocytes were Siglecf+ macrophages (CD45+/Siglecf+CD11c^high^). Pro-inflammatory macrophage recruitment is dampened by MPLA alone (~30% total BAL cells), with the most significant dampening observed after vaccination with the O-glycan-restricted RSV G^ecto^ SC GALNT3 protein (*p* = 0.0001) (Figure 6A). The study indicated a localized enhanced presence of mature antibody-secreting B220+ B cells in all mice administered MPLA (*p* = 0.05), with a notable increase in lung-resident mature B cells in mice vaccinated with O-glycan-restricted RSV G^ecto^ SC GALNT3 (*p* = 0.0001) (Figure 6B). There was no substantive Siglecf+ eosinophilic infiltrate (CD45+/Siglecf+CD11c^low^) (Figure 6C), and unusually few infiltrating T lymphocytes were detected (Figure 6D).

## 4. Discussion

To determine if the RSV vaccine response was modified by restricting O-linked glycosylation on an RSV G^ecto^ glycoprotein vaccine, we examined the outcome in a preliminary BALB/c mouse challenge study, challenging with a virus with an identical RSV G sequence that was used for immunization. RSV G^ecto^ immunogens with identical amino acid sequence but differing O-linked glycosylation were expressed and purified from three CHO cell lines. Comparing O-glycosylation-restricted RSV G^ecto^ proteins by electrophoretic mobility by SDS-PAGE, we observed that only approximately half of the predicted O-linked glycosylation sites on RSV G^ecto^ are occupied by GalNAc in the absence of GALNT3. This finding emphasizes the reason to carefully examine O-glycosylation when producing recombinant viral glycoproteins with mucin-like domains as vaccine antigens.

We subsequently vaccinated six groups of BALB/c mice (Figure 2) with one of each of the three RSV G^ecto^ proteins (50 µg) adjuvanted with MPLA, followed by an identical boost three weeks later. The vaccinated mice were challenged with 10^6^ PFU RSV/A2 a week after the vaccination regime was completed. Control groups included naïve, MPLA-immunized, and infection-only controls. O-glycosylation-restricted RSV G^ecto^ proteins were significantly more immunogenic than non-restricted O-glycosylated RSV G^ecto^. Even a single dose of O-glycosylation-restricted RSV G^ecto^ vaccine antigen raised a moderate anti-RSV G CCD response. Post boost, a circulating anti-RSV G^ecto^ IgG endpoint titer of 10^5^ was attained compared to a mean titer of 5 × 10^3^ by mice vaccinated with a non-restricted RSV G^ecto^ protein. Only the O-glycosylation-restricted RSV G^ecto^ protein immunized groups achieved statistical significance (*p* = 0.0001) and despite the immunogenicity of both RSV G^ecto^ SC and SC GALNT3 proteins, only vaccination with RSV G^ecto^ SC GALNT3 protein induced autologous-virus-specific neutralizing antibodies. This could be due to the requirement for a very particular configuration of GalNAc loaded residues to expose neutralization epitopes, and/or due to differences in proteolysis due to the more extensive glycan occupancy of RSV G^ecto^ SC GALNT3.

In concordance with the detection of neutralizing antibodies, the RSV G^ecto^ SC GALNT3 protein-vaccinated group of mice had significantly reduced lung viral load, five days after challenge with autologous virus. RSV G^ecto^ proteins, when administered in combination with the TLR4 agonist MPLA, had no significant effect on post-vaccination pulmonary infiltrate in our BALB/c experiment.

Precedent for the manipulation of O-linked glycans in vaccine antigen design. Recently, the improved efficacy of a currently available CHO-produced recombinant Herpes Zoster glycoprotein gE subunit vaccine was determined to be due to altered exposure of a B cell epitope that is shielded by O-linked glycosylation when the vaccine antigen is produced in a different cell line [92]. There is also precedent for the elimination of N- and O-linked glycans in the generation of RSV G^ecto^ vaccine antigens via protein expression in *E. coli* [90]. However, this protein was produced in an insoluble form and then refolded in the presence of a redox buffer, so it is unclear if this antigen displays conformational epitopes like those for mAbs 3D3 and 2D10. Even so, this non-glycosylated RSV G^ecto^ vaccine antigen was found to be significantly more immunogenic than the fully glycosylated protein, eliciting neutralizing antibodies in both BALB/c mice and cotton rats [90,93]. Furthermore, the non-glycosylated RSV G^ecto^ has antigenic epitopes outside of the central conserved domain, which may contribute towards a protective anti-RSV response [94].

Here, by employing the SimpleCell strategy to produce soluble RSV G^ecto^ antigens, the conformational epitopes in the CCD are retained, sites previously masked by O-linked core polysaccharide extension may be de-shielded, and new epitopes added. This is a new use of the SimpleCell technology, which was originally developed to produce O-linked glycoproteins with homogeneous O-glycosylation to map the O-glycoproteome by mass spectrometry. We found that O-glycan-restricted RSV G^ecto^ glycoproteins appear to be more effective in raising antibodies to full-length RSV G than a non-restricted protein when used in combination with MPLA to vaccinate BALB/c mice. All RSV G^ecto^ proteins were effective in raising a response to the non-glycosylated and highly conserved CCD, but again, only mice immunized with the O-glycan-restricted RSV G^ecto^ glycoproteins demonstrated a titer with a statistical confidence higher than MPLA vaccination alone. Vaccination with RSV G^ecto^ SC GALNT3 protein raised antibodies capable of neutralizing autologous virus in vitro and reducing lung viral load in vivo. Preliminary examination of inflammatory lung infiltrate post challenge suggests that vaccine antigens were well-tolerated in this experiment. Future work should include examining the theoretical mechanisms of vaccine-enhanced disease (VED), including antibody-enhanced disease and Th2-mediated pathology. It should be noted that this is a preliminary pilot study and the first use of a GalNAc-enriched viral glycoprotein as a vaccine antigen in vivo. The cotton rat model may be better to mimic RSV pathology and potential VED, and examination of cross-reactivity to other RSV A or B strains might be justified. GalNAc proteoglycan B cell epitopes have been identified on other viruses [61,67,68]. They are known to be immunogenic, but these antigens are thought to be rapidly cleared from serum due to their high affinity for liver-expressed asialoglycoprotein receptor [95].

The production of viral glycoproteins in differing cell types with differing capacity to initiate and extend core O-linked glycans is an underexplored field. Our study supports the importance in further exploring the manipulation of O-linked glycosylation to enhance the efficacy of RSV G^ecto^ as a vaccine antigen.

## Figures and Tables

**Table 1:** Binding affinity constant (K_D_), standard deviation (K_D_ SD), on-rates (k_a_), off-rates (k_d_), R^2^, and x^2^ of RSV G^ecto^ immunogens to conformation-dependent mAbs 3D3 and 2D10.

	mAb	K_D_ (pM) *	K_D_ SD (pM)	k_a_ (10^5^ M^−1^s^−1^)	k_d_ (10^−7^s^−1^)	R^2^	x^2^
CHO-S G^ecto^	3D3	<1.0	3.0	1.00	<1	0.9927	0.0562
CHO-S G^ecto^	2D10	<1.0	1.7	5.00	<1	0.9947	0.1022
SC GALNT3 G^ecto^	3D3	1.0	<1.0	6.00	<1	0.9906	0.0667
SC GALNT3 G^ecto^	2D10	<1.0	<1.0	2.00	<1	0.9943	0.0468
SC G^ecto^	3D3	<1.0	<1.0	8.00	<1	0.9738	0.1689
SC G^ecto^	2D10	2.0	1.6	5.00	<1	0.9888	0.0300

* Average K_D_ values are reported as the average of the three replicate binding studies.

## Data Availability

Experimental data is deposited at https://datadryad.org.
